# Performance Analysis of Diversity-Controlled Multi-User Superposition Transmission for 5G Wireless Networks

**DOI:** 10.3390/s18020536

**Published:** 2018-02-10

**Authors:** Jeong Seon Yeom, Eunmi Chu, Bang Chul Jung, Hu Jin

**Affiliations:** 1 Department of Electronics Engineering, Chungnam National University, Daejeon 34134, Korea; jsyeom@cnu.ac.kr (J.S.Y.); emchu@cnu.ac.kr (E.C.); 2 Division of Electrical Engineering, Hanyang University, Ansan 15588, Korea

**Keywords:** 5G wireless networks, non-orthogonal multiple access (NOMA), OFDM, frequency diversity, multiple antennas, error probability

## Abstract

In this paper, we propose a novel low-complexity multi-user superposition transmission (MUST) technique for 5G downlink networks, which allows multiple cell-edge users to be multiplexed with a single cell-center user. We call the proposed technique *diversity-controlled MUST technique* since the cell-center user enjoys the frequency diversity effect via signal repetition over multiple orthogonal frequency division multiplexing (OFDM) sub-carriers. We assume that a base station is equipped with a single antenna but users are equipped with multiple antennas. In addition, we assume that the quadrature phase shift keying (QPSK) modulation is used for users. We mathematically analyze the bit error rate (BER) of both cell-edge users and cell-center users, which is the first theoretical result in the literature to the best of our knowledge. The mathematical analysis is validated through extensive link-level simulations.

## 1. Introduction

Fifth generation (5G) wireless networks are expected to support higher spectral efficiency, lower end-to-end latency, and more connection nodes [1]. In accordance with this trend, many promising techniques are being considered [2]. Among them, a non-orthogonal multiple access (NOMA) technique has been proposed to improve the spectral efficiency of the 5G network [3]. The basic idea of the NOMA technique is to serve multiple users at the same time-frequency-space resource block via power domain or code domain multiplexing [4]. It has been known that the NOMA technique increases the spectrum efficiency and efficiently accommodates a massive number of nodes in cellular networks [5,6].

As noted before, the NOMA techniques are, in general, classified into two categories: code-domain NOMA and power-domain NOMA [7]. With the code-domain NOMA techniques, a codeword is allocated to each user and a near optimal multi-user detection algorithm, such as a message passing algorithm, is used at the receiver. The code-domain NOMA techniques include trellis-coded multiple access (TCMA), interleave-division multiple access (IDMA), low-density signature (LDS) sequence-based code division multiple access (CDMA), sparse-code multiple access (SCMA), pattern-division multiple access (PDMA), multi-user shared access (MUSA), etc. [8]. On the other hand, with the power domain NOMA techniques, multiple users are served within a given time-frequency-space resource block by using superposition coding (SC) at the transmitter and successive interference cancellation (SIC) at the receivers, respectively, which has recently been proposed in 3GPP LTE [3].

Multi-user superposition transmission (MUST) is a special case of the NOMA techniques, which has been studied in 3GPP LTE standards [9]. Various NOMA techniques have been proposed and studied, focusing on multi-user non-orthogonal transmission schemes, receiver designs, and related signaling strategies in [9]. The MUST techniques are divided into three categories according to adaptive power control and bit-labeling at the transmitter side as shown in Table 1. The adaptive power ratio on each component constellation is utilized in MUST categories 1 and 2, and Gray-mapped composite constellation is adopted in MUST categories 2 and 3. The MUST techniques typically assume asymmetric downlink scenarios consisting of active cell-edge and cell-center users. At the receiver side, the cell-center user with higher received signal power decodes a super-imposed signal with SIC, while the cell-edge user with lower received signal power decodes the super-imposed signal by treating the interference signal as noise. To implement SIC at the cell-center user, codeword-level (channel coding block) SIC results in better performance than symbol-level SIC, but the signaling overhead and implementation complexity of the codeword-level SIC is much higher that of the symbol-level SIC [10,11]. Thus, the codeword-level SIC may not be suitable in the cellular downlink, especially when the low-cost and low-power user terminals/devices are considered.

Recently, several receiver designs of the NOMA techniques, including codeword-level SIC and symbol-level SIC, have been compared with each other. In particular, a log-likelihood ratio (LLR)-based low-complexity receiver design was proposed [12]. Although the LLR-based receiver design does not include the codeword-level SIC operation which accompanies high implementation complexity, it can still achieve similar performance to the codeword-level SIC receiver as well as the ideal SIC receiver. However, the performance of the proposed technique was evaluated only through link-level simulations. In addition, another low-complexity MUST technique was proposed in [13], where a NOMA transmitter sends signals to a single cell-center user over multiple (frequency) resource blocks, each of which is also occupied by a cell-edge user. Thus, the cell-center user can obtain the frequency diversity, and we call this scheme *diversity-controlled MUST* technique in this paper. However, in [13], the rate outage performance was mathematically analyzed when there exist only two cell-edge users and each user is equipped with a single receive antenna.

In this paper, we extend the diversity-controlled MUST technique to the case where multiple cell-edge users are multiplexed with a single cell-center user and each user is equipped with multiple antennas. Henceforth, we define the user with a higher signal-to-noise ratio (SNR) as *MUST-based user equipment* (UE) and the user with a lower SNR as *MUST-enhanced UE* since the received SNR depends on not only the location of users but also the allocated power in the diversity-controlled MUST technique. In general, cell-center users become the MUST-based UEs and cell-edge users become the MUST-enhanced UEs. In particular, we derive the closed-form expressions on the bit error rate (BER) of both MUST-based UEs and MUST-enhanced UEs in Rayleigh fading channels for a given power allocation ratio. Based on the mathematical analysis, we also optimize the power allocation ratio on the component constellation to minimize the power consumption at the NOMA transmitter for given BER requirements. The mathematical analysis is validated via link-level simulations.

The remainder of the paper is organized as follows. In Section 2, the system model is described; in Section 3, we explain the overall procedures of both the transmitter and receiver with the proposed diversity-controlled MUST technique in detail and derive the BER of the proposed technique; in Section 4, we formulate an optimization problem of the power allocation ratio to minimize the power consumption at the NOMA transmitter; simulation results are shown in Section 5; finally, conclusions are drawn in Section 6.

## 2. System Model

We consider a downlink cellular network consisting of a single base station (BS), a single MUST-enhanced UE, and *N* MUST-based UEs. We assume that the BS is equipped with a single transmit antenna but each user is equipped with $N_{r}$ antennas. Hence, the wireless channel from the BS to each user is modeled as a single-input multiple-output (SIMO) channel. We consider that the orthogonal frequency division multiple access (OFDMA) and the system bandwidth are assumed to be equally divided into *N* sub-carriers for simplicity.

**Figure 1:**
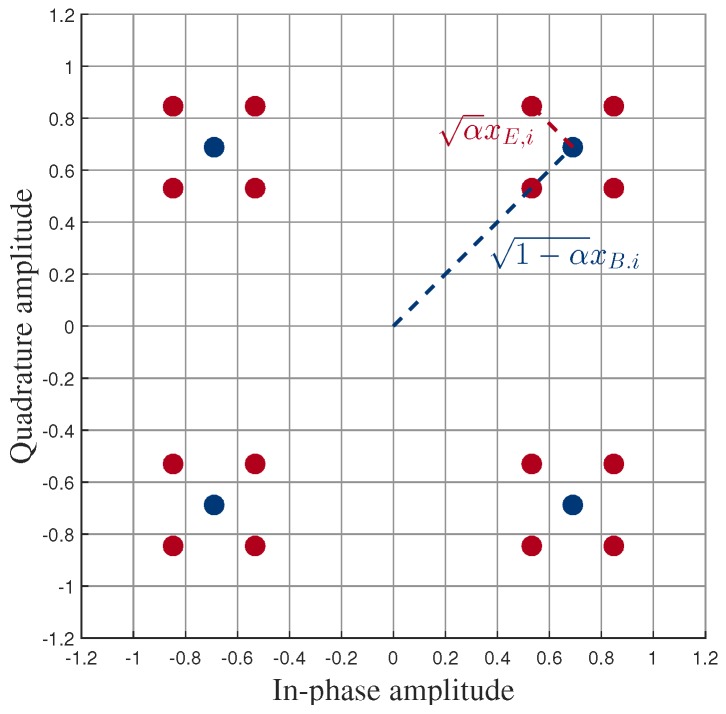
Example of the composite constellation of the MUST technique with quadrature phase shift keying (QPSK) modulation.

At the BS, a composite modulation symbol is generated by superposing two signals of an MUST-based UE and an MUST-enhanced UE. We adopt the MUST category 2 when generating the composite symbol, and thus the power ratio between two component signals can be adaptively adjusted and Gray-mapping is used for bit-labeling. An adaptive power allocation coefficient, α(0<α<0.5), is introduced to determine powers of the two signals. Hence, the α value needs to be carefully adjusted, depending on the channel conditions of users. Each superposed signal is transmitted to both the MUST-based UE and the MUST-enhanced UE over a certain sub-band. Figure 1 shows an example of a composite constellation of the MUST technique with quadrature phase shift keying (QPSK) modulation for both users. The blue dots represent QPSK constellation points of the MUST-based UE whose power is equal to 1−α and the red dots around each blue dot represent the composite constellation points, consisting of QPSK constellations of both MUST-based and MUST-enhanced UEs, whose power is equal to 1. As shown in Figure 1, α indicates the power portion that is allocated to the MUST-enhanced UE. As noted before, the signal of the MUST-enhanced UE is transmitted over multiple frequency bands (sub-carriers), while the signal of the MUST-based UE is sent over a single frequency band (sub-carrier) with the diversity-controlled MUST technique [13].

At the MUST-based UE, the received signal is decoded by treating the signal of the MUST-enhanced UE as noise. On the other hand, at the MUST-enhanced UE, the received signal is obtained after the symbol-level SIC operation for each frequency band. Then, the obtained signals over multiple frequency bands (sub-carriers) are combined to obtain the frequency diversity gain.

## 3. Diversity-Controlled MUST Technique

In Section 3.1, we explain the transmitter design of the proposed diversity-controlled MUST technique including the bit-labeling and the power allocation at the BS. In Section 3.2 and Section 3.3, we explain the receiver designs for the MUST-based UE and the MUST-enhanced UE and mathematically analyze the BER of the two types of UEs, respectively.

### 3.1. Transmitter Design at the BS

**Figure 2:**
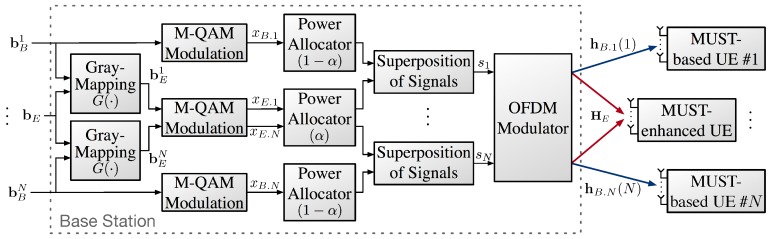
Transmitter design of the diversity-controlled MUST technique.

Figure 2 shows the overall structure of the transmitter at the BS in the proposed diversity-controlled MUST technique. Bit-streams $b_{B}^{i}=\left\{b_{B.1}^{i},\ldots ,b_{B.j}^{i},\ldots ,b_{B.m}^{i}\right\}$ and $b_{E}=\left\{b_{E.1},\ldots ,b_{E.j},\ldots ,b_{E.m}\right\}$ enter to the BS, where *M* denotes the modulation order, i.e., $m=\log_{2}M$. Bit-streams $b_{B}^{i}$ and $b_{E}$ denote the bit-streams for the *i*-th MUST-based UE $\left(i\in \{1,2,\ldots ,N\}\right)$ and the MUST-enhanced UE, respectively. The term *j* indicates the *j*-th bit in an *M*-QAM symbol. According to the bit-labeling rule (Gray-mapping) of the MUST category 2, the bits of the MUST-enhanced UE are coded by the following function, G(·):

(1) $$G\left(b_{E.j},b_{B.j}^{i}\right)=\left(b_{E.j}⊕b_{B.j}^{i}\right)=b_{E.j}^{i},$$

where ⊕ denotes a bitwise XOR operator. The Gray-mapped bit-streams $b_{B}^{i}$ and $b_{E}^{i}$ are then modulated by *M*-QAM. The modulated symbols to be sent via the *i*-th sub-carrier for the MUST-based UE and the MUST-enhanced UE are denoted by $x_{B.i}$ and $x_{E.i}$, respectively. The $x_{B.i}$ and $x_{E.i}$ are assigned power by 1−α and α, respectively. In this paper, we assume that α is the same value for all *i*. Thus, the super-imposed signal in the *i*-th sub-carrier, $s_{i}$, is given by

(2) $$s_{i}=\sqrt{1-\alpha }x_{B.i}+\sqrt{\alpha }x_{E.i},$$

where $E[|x_{B.i}{|}^{2}]=E[|x_{E.i}{|}^{2}]=1$. Thus, $E[|s_{i}{|}^{2}]=1$.

### 3.2. Receiver Design at an MUST-Based UE

**Figure 3:**
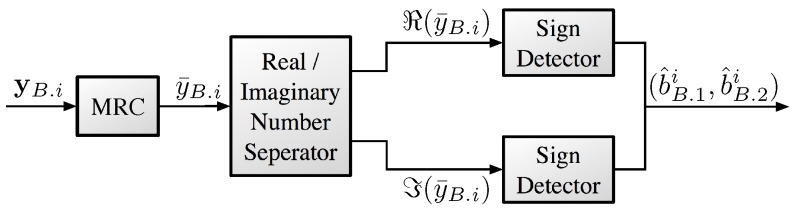
Receiver structure at the MUST-based UE with the diversity-controlled MUST technique.

Figure 3 shows the overall structure of the proposed receiver at the MUST-based UE. Recall that the signal of the *i*-th MUST-based UE is assumed to be sent via the *i*-th OFDM sub-carrier. Then, the received signal of the *i*-th MUST-based UE with $N_{r}$ antennas is given by

(3) $$y_{B.i}=h_{B.i}\left(i\right)\sqrt{E}s_{i}+w_{B.i},$$

where E indicates the transmit power of $s_{i}$ and $h_{B.i}\left(i\right)\in C^{N_{r}\times 1}$ denotes the wireless channel vector from the BS to the *i*-th MUST-based UE. We assume that E is a fixed value over frequencies but it can be adaptively controlled to satisfy a certain performance requirement. We assume that each element of $h_{B.i}\left(i\right)$ follows identically and independently distributed (i.i.d.) complex-valued normal Gaussian distribution, i.e., $h_{B.i}\left(i\right)∼\mathrm{CN}\left(0,I_{N_{r}\times N_{r}}\right)$, where $I_{N_{r}\times N_{r}}$ indicates the identity matrix of dimension $N_{r}$. Furthermore, $w_{B.i}\in C^{N_{r}\times 1}$ denotes the additive white Gaussian noise (AWGN) vector at the *i*-th MUST-based UE, i.e., $w_{B.i}∼\mathrm{CN}\left(0,N_{0}I_{N_{r}\times N_{r}}\right)$, where $N_{0}$ indicates the power spectral density of the Gaussian noise.

With maximum ratio combining, the received signal vector at the MUST-based UE can be represented as

$$\begin{array}{cc} {\bar{y}}_{B.i}= & h_{B.i}{\left(i\right)}^{H}y_{i}\\ = & {∥h_{B.i}\left(i\right)∥}^{2}\sqrt{E}s_{i}+h_{B.i}{\left(i\right)}^{H}w_{B.i}\\ = & {∥h_{B.i}\left(i\right)∥}^{2}\sqrt{E}\left(\sqrt{\alpha -1}x_{B.i}+\sqrt{\alpha }x_{E.i}\right)+h_{B.i}{\left(i\right)}^{H}w_{B.i}, \end{array}$$

where $A^{H}$ denotes the conjugate transpose matrix of A. Let $W_{B.i}≜h_{B.i}{\left(i\right)}^{H}w_{B.i}$. Then, due to properties of the circular symmetric Gaussian random vector [11], $W_{B.i}$ can be represented by

(4) $$W_{B.i}∼\mathrm{CN}\left(0,N_{0}\right).$$

Assuming that α<0.5, $x_{B.i}$ determines the signs of $s_{i}$ as shown in Figure 1. In other words, $Sgn\left(ℜ\left(x_{B.i}\right)\right)$ and $Sgn\left(ℑ\left(x_{B.i}\right)\right)$ equal to $Sgn\left(ℜ\left(s_{i}\right)\right)$ and $Sgn\left(ℑ\left(s_{i}\right)\right)$, respectively, where Sgn(·) denotes the sign function. If $b_{B.1}^{i}=1$, then

(5) $$ℜ\left(s_{i}\right)\in \left\{\sqrt{\frac{1}{2}}\left(\sqrt{1-\alpha }-\sqrt{\alpha }\right),\sqrt{\frac{1}{2}}\left(\sqrt{1-\alpha }+\sqrt{\alpha }\right)\right\}:=\left\{d_{1},d_{2}\right\}$$

according to the transmitter design. Thus, the decoding function for $b_{B.j}^{i}$ from ${\bar{y}}_{B.i}$ is given as:

(6) $$\left({\hat{b}}_{B.1}^{i},{\hat{b}}_{B.2}^{i}\right)=M^{-1}\left(Sgn\left(ℜ\left({\bar{y}}_{B.i}\right)\right),Sgn\left(ℑ\left({\bar{y}}_{B.i}\right)\right)\right),$$

where ${\hat{b}}_{B.1}^{i}$ and ${\hat{b}}_{B.2}^{i}$ denote the estimated bits for $b_{B.1}^{i}$ and $b_{B.2}^{i}$, respectively. $M^{-1}\left(\cdot \right)$ represents the QPSK demodulation function.

Table 2 summarizes the computation complexity of the proposed receiver at the MUST-based UE in terms of the complex number of operations.

### 3.3. Performance Analysis of the MUST-Based UE

Without loss of generality, the error probability of the MUST-based UE for given $h_{B.i}\left(i\right)$ is represented as:

(7) Pb.B=Prb^B.1i=0|bB.1i=1,hB.i(i)=∑d∈{d1,d2}Prℜ(si)=d|bB.1i=1Prℜ(y¯B.i)|ℜ(si)=d,hB.i(i)=∑d∈{d1,d2}12∫−∞012πN02EhB.i(i)2exp−(x−d)22N02EhB.i(i)2dx=∑d∈{d1,d2}12∫−∞−2EN0hB.i(i)2d12πexp−x22dx=∑d∈{d1,d2}12Q2ρhB.i(i)2d2,

where $\rho ≜\frac{E}{N_{0}}$ indicates the transmit SNR at the BS.

The average BER of the MUST-based UE can be obtained by integrating Equation (7) over the random variable (RV) ${∥h_{B.1}\left(i\right)∥}^{2}$. Note that the RV ${∥h_{B.1}\left(i\right)∥}^{2}$ follows the Chi-square distribution with $2N_{r}$ degrees-of-freedom (DoF) [11], i.e., $Z_{i}≜{∥h_{B.i}\left(i\right)∥}^{2}∼\chi_{2N_{r}}^{2}$.

Then, the average BER of the MUST-based UE is given as:

(8) P¯b.B=∫0∞Pb.BfZi(zi)dzi=∫0∞∑d∈{d1,d2}12Q2ρzid2fZi(zi)dzi=12∑d∈{d1,d2}∫−∞∞Q2ρzid21Nr−1!ziNr−1exp−zidzi=∑d∈{d1,d2}1−d2ρ1+d2ρNr∑i=0Nr−1Nr−1+ii12Nr+1+i1+d2ρ1+d2ρi,

where $d_{1}$ and $d_{2}$ are defined in Equation (5).

### 3.4. Receiver Design at an MUST-Enhanced UE

**Figure 4:**
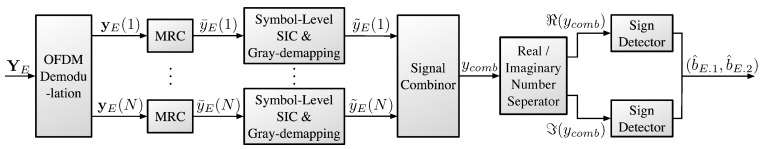
Receiver structure at the MUST-enhanced UE with the diversity-controlled MUST technique.

Figure 4 shows the overall structure of the proposed receiver at the MUST-enhanced UE. Unlike the signals at the MUST-based UEs, the signal of the MUST-enhanced UE is superposed over *N* MUST-based signals since it is repeatedly sent over *N* OFDM sub-carriers. Thus, in order for the MUST-enhanced UE to obtain full diversity gain, we need to combine all the signals over *N* OFDM sub-carriers and in $N_{r}$ receive antennas. The received signal at the MUST-enhanced UE is given by

(9) $$Y_{E}=H_{E}\sqrt{E}diag\left(s\right)+W_{E},$$

where $Y_{E}≜\left[\begin{array}{cccc} y_{E}\left(1\right) & y_{E}\left(2\right) & \cdots & y_{E}\left(N\right) \end{array}\right]\in C^{N_{r}\times N}$. A vector $y_{E}\left(i\right)$ denotes the signal vector received at the *i*-th OFDM sub-carrier of the MUST-enhanced UE. A matrix $H_{E}≜\left[\begin{array}{cccc} h_{E}\left(1\right) & h_{E}\left(2\right) & \cdots & h_{E}\left(N\right) \end{array}\right]\in C^{N_{r}\times N}$ denotes the wireless channel matrix from the BS to the MUST-enhanced UE, where each element follows i.i.d. complex-valued normal Gaussian distribution and $h_{E}\left(i\right)$ denotes the channel vector of the $s_{i}$ transmitted over the *i*-th OFDM sub-carrier. A matrix diag(s) indicates the diagonal matrix whose diagonal elements form the vector s. In addition, $s≜\left[\begin{array}{cccc} s_{1} & s_{2} & \cdots & s_{N} \end{array}\right]\in C^{1\times N}$ indicates the transmit signal vector. A matrix $W_{E}=\left[\begin{array}{cccc} w_{E}\left(1\right) & w_{E}\left(2\right) & \cdots & w_{E}\left(N\right) \end{array}\right]\in C^{N_{r}\times N}$ represents the additive white Gaussian noise matrix whose element is a zero mean complex Gaussian RV with $N_{0}$ variance, and $w_{E}\left(i\right)$ denotes the noise vector in the received signal vector over the *i*-th OFDM sub-carrier. Each column of $Y_{E}$ can be separated and dealt with independently over *N* sub-carriers. Then, the received signal vector over the *i*-th sub-carrier at the MUST-enhanced UE is given by

(10) $$y_{E}\left(i\right)=h_{E}\left(i\right)\sqrt{E}s_{i}+w_{E}\left(i\right).$$

Similarly to ${\bar{y}}_{B.i}$, after MRC, the received signal vector in the *i*-th sub-carrier at the MUST-enhanced UE is given by

(11) $${\bar{y}}_{E}\left(i\right)={∥h_{E}\left(i\right)∥}^{2}\sqrt{E}\left(\sqrt{\alpha -1}x_{B.i}+\sqrt{\alpha }x_{E.i}\right)+h_{E}{\left(i\right)}^{H}w_{E}\left(i\right).$$

The receiver of the MUST-enhanced UE first detects $x_{B.i}$ like the receiver operation of the MUST-based UE, and then removes ${\hat{x}}_{B.i}$ from the ${\bar{y}}_{E}\left(i\right)$, which is called symbol-level SIC operation in the literature. Considering the Gray-mapping function $G\left(\cdot \right)$, Equation (11) can be rewritten as:

(12) $$\begin{array}{cc} {\tilde{y}}_{E}\left(i\right)= & \left[ℜ\left({\bar{y}}_{E}\left(i\right)\right)-\underset{\mathrm{SIC}}{\underset{︸}{{∥h_{E}\left(i\right)∥}^{2}\sqrt{E}\sqrt{1-\alpha }Sgn\left(ℜ\left({\bar{y}}_{E}\left(i\right)\right)\right)}}\right]\cdot \underset{\mathrm{Gray}-\mathrm{demapping}}{\underset{︸}{Sgn\left(ℜ\left({\bar{y}}_{E}\left(i\right)\right)\right)}}\\ & +j\left[ℑ\left({\bar{y}}_{E}\left(i\right)\right)-\underset{\mathrm{SIC}}{\underset{︸}{{∥h_{E}\left(i\right)∥}^{2}\sqrt{E}\sqrt{1-\alpha }Sgn\left(ℑ\left({\bar{y}}_{E}\left(i\right)\right)\right)}}\right]\cdot \underset{\mathrm{Gray}-\mathrm{demapping}}{\underset{︸}{Sgn\left(ℑ\left({\bar{y}}_{E}\left(i\right)\right)\right)}}. \end{array}$$

By summing Equation (12) over *N* sub-carriers, the final received signal for the MUST-enhanced UE can be obtained as follows:

(13) $$y_{comb}=\sum_{i=1}^{N}{\tilde{y}}_{E}\left(i\right).$$

If the SIC is successfully executed in Equation (12), then ${\tilde{y}}_{E}\left(i\right)$ is given by

(14) $${\tilde{y}}_{E}\left(i\right)={∥h_{E}\left(i\right)∥}^{2}\sqrt{E}\sqrt{\alpha }x_{E}+h_{E}{\left(i\right)}^{H}w_{E}\left(i\right),$$

where $x_{E}$ is the QPSK symbol mapped from the information bit pair $\left(b_{E.1},b_{E.2}\right)$. In addition, with the perfect SIC, the final received signal for the MUST-enhanced UE can be obtained as follows:

(15) $$y_{comb}=\sum_{i=1}^{N}{\tilde{y}}_{E}\left(i\right)=\mathrm{Tr}\left(H_{E}^{H}H_{E}\right)\sqrt{E}\sqrt{\alpha }x_{E}+\sum_{i=1}^{N}h_{E}{\left(i\right)}^{H}w_{E}\left(i\right),$$

where $\mathrm{Tr}\left(\cdot \right)$ denotes the trace operator of the matrix. Let $W_{comb}≜\sum_{i=1}^{N}h_{E}{\left(i\right)}^{H}w_{E}\left(i\right)$ and then $W_{comb}$ can be written as:

(16) $$W_{comb}∼\mathrm{CN}\left(0,N\cdot N_{0}\right).$$

The information bit pair $\left(b_{E.1},b_{E.2}\right)$ can be decoded by the sign of $y_{comb}$ as follows:

(17) $$\left({\hat{b}}_{E.1},{\hat{b}}_{E.2}\right)=M^{-1}\left(Sgn\left(ℜ\left(y_{comb}\right)\right),Sgn\left(ℑ\left(y_{comb}\right)\right)\right),$$

where ${\hat{b}}_{E.1}$ and ${\hat{b}}_{E.2}$ denote the estimates of $b_{E.1}$ and $b_{E.2}$, respectively.

Table 3 summarizes the computation complexity of the proposed receiver at the MUST-enhanced UE in terms of the complex number of operations. Basically, the computational complexity at the MUST-enhanced UE is larger than that at the MUST-based UE because of the symbol-level SIC operation and the signal transmission over multiple sub-carriers.

### 3.5. Performance Analysis of the MUST-Enhanced UE

The error events of the MUST-enhanced UE happen with the following two cases:

- Incorrect detection of both the symbol $x_{E.i}$ and the bit $b_{B.1}$.
- Accurate detection of the symbol $x_{E.i}$ but incorrect detection of the bit $b_{B.1}$.

Thus, the BER of the MUST-enhanced UE is given as:

(18) $$\begin{array}{cc} P_{b.E}= & \mathrm{Pr}\left\{\left({\hat{b}}_{B.1}^{i},{\hat{b}}_{B.2}^{i}\right)\neq \left(b_{B.1}^{i},b_{B.2}^{i}\right),{\hat{b}}_{E.1}\neq b_{E.1}\right\}+\mathrm{Pr}\left\{\left({\hat{b}}_{B.1}^{i},{\hat{b}}_{B.2}^{i}\right)=\left(b_{B.1}^{i},b_{B.2}^{i}\right),{\hat{b}}_{E.1}\neq b_{E.1}\right\}\\ = & \mathrm{Pr}\left\{\left({\hat{b}}_{B.1}^{i},{\hat{b}}_{B.2}^{i}\right)\neq \left(b_{B.1}^{i},b_{B.2}^{i}\right)\right\}\mathrm{Pr}\left\{{\hat{b}}_{E.1}\neq b_{E.1}|\left({\hat{b}}_{B.1}^{i},{\hat{b}}_{B.2}^{i}\right)\neq \left(b_{B.1}^{i},b_{B.2}^{i}\right)\right\}\\ & \;+\mathrm{Pr}\left\{\left({\hat{b}}_{B.1}^{i},{\hat{b}}_{B.2}^{i}\right)=\left(b_{B.1}^{i},b_{B.2}^{i}\right)\right\}\mathrm{Pr}\left\{{\hat{b}}_{E.1}\neq b_{E.1}|\left({\hat{b}}_{B.1}^{i},{\hat{b}}_{B.2}^{i}\right)=\left(b_{B.1}^{i},b_{B.2}^{i}\right)\right\}. \end{array}$$

Since the error probability of decoding $\left(b_{B.1}^{i},b_{B.2}^{i}\right)$ is close to zero at high SNR values, we can obtain the lower bound of the BER by assuming that the decoding on $\left(b_{B.1}^{i},b_{B.2}^{i}\right)$ is always correct:

(19) $$P_{b.E}\geq \mathrm{Pr}\left\{{\hat{b}}_{E.1}\neq b_{E.1}|\left({\hat{b}}_{B.1}^{i},{\hat{b}}_{B.2}^{i}\right)=\left(b_{B.1}^{i},b_{B.2}^{i}\right)\right\}≜P_{b.E}^{LB}.$$

Without loss of generality, the lower bound of the BER of the MUST-enhanced UE with this assumption for a given $H_{E}$ is obtained as:

(20) Pb.ELB=Prb^E.1=0∣bE.1=1,b^B.1i,b^B.2i=bB.1i,bB.2i,HE=Prℜycomb≤0∣bE.1=1,b^B.1i,b^B.2i=bB.1i,bB.2i,HE=Prℜycomb≤0∣ℜxE=1,b^B.1i,b^B.2i=bB.1i,bB.2i,HE=∫−∞012πN02EαTrHEHHEexp−x−122N02EαTrHEHHEdx=∫−∞−2EN0αTrHEHHE12πexp−x22dx=Q2ραTrHEHHE.

We define an RV as follows:

(21) $$Z≜\mathrm{Tr}\left(H_{E}^{H}H_{E}\right)∼\chi_{2N\cdot N_{r}}^{2}.$$

Then, the lower bound of the average BER of the MUST-enhanced UE is given by

(22) P¯b.ELB=∫0∞Pb.ELBfZzdz=∫0∞Q2ραz1Nr·N−1!zNr·N−1exp(−z)dz=1−ρα1+ραNr·N∑k=0Nr·N−1Nr·N−1+kk12Nr+k1+ρα1+ραk.

## 4. Optimal α for Minimizing Power Consumption

In Equations (8) and (22), the power allocated to each user’s signal determines its point-to-point BER performance. Of cause, using a lot of power may guarantee the BER requirements of the users. However, the available energy of the BS is limited in general, and thus it is necessary to use the power efficiently. This can be accomplished by transmitting the signal with the minimum power at the BS while satisfying the required BER performance of each user. For given BER requirements for users, there exists the optimal power allocation coefficient, α, to minimize the required energy, E, at the BS. In general, the BER performance of the MUST-enhanced UE becomes improved as α increases for a given E, while that of the MUST-based UE becomes improved as α decreases for a given E. Thus, there exists a trade-off between the BER performances of the MUST-based UE and the MUST-enhanced UE according to α.

In this section, we formulate the optimization problem to minimize the power consumption at the BS for given BER requirements for users. $E_{B}\left(\alpha ,P_{b.B}^{req}\right)$ is defined as the required power at the BS to satisfy the BER requirement of the MUST-based UE $P_{b.B}^{req}$ for a given α. Similarly, $E_{E}\left(\alpha ,P_{b.E}^{req}\right)$ is defined as the required power at the BS to satisfy the BER requirement of the MUST-enhanced UE $P_{b.E}^{req}$ for a given α. Then, the optimal power allocation coefficient to minimize the power consumption of the BS is given as:

(23) $$\begin{array}{c} \alpha^{*}=\underset{\alpha }{\arg}\min\left[\max\left(E_{B}\left(\alpha ,P_{b.B}^{req}\right),E_{E}\left(\alpha ,P_{b.E}^{req}\right)\right)\right]\\ s.t.\;\;0<\alpha <0.5. \end{array}$$

The optimization problem shown in Equation (23) can be solved easily as follows. For given BER requirements $P_{b.B}^{req}$ and $P_{b.E}^{req}$, $E_{B}\left(\alpha ,P_{b.B}^{req}\right)$ is a monotonically increasing function α, while $E_{E}\left(\alpha ,P_{b.E}^{req}\right)$ is a monotonically decreasing function of α since α denotes the power portion allocated to the MUST-enhanced UE. Thus, the optimal power allocation coefficient $\alpha^{*}$ can be obtained by solving the following equation:

(24) $$E_{B}\left(\alpha^{*},P_{b.B}^{req}\right)=E_{E}\left(\alpha^{*},P_{b.E}^{req}\right).$$

## 5. Simulation Results

In this section, we show the performance of the proposed diversity-controlled MUST technique. In particular, we evaluate the average BER performances of both the MUST-based UE and the MUST-enhanced UE. We show the minimum required power at the BS for satisfying the BER requirement according to α. In all link-level simulations, we utilize QPSK modulation and Rayleigh fading channels.

**Figure 5:**
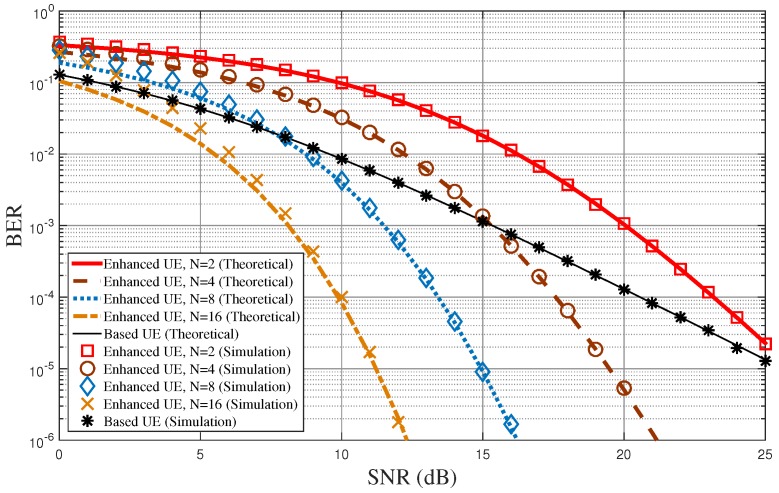
BER performance of the proposed MUST technique for varying SNR values when α=0.05, N=2,4,8,16, and $N_{r}=2$.

Figure 5 shows the BER performance of the proposed diversity-controlled MUST technique for varying SNR values when α=0.05, N=2,4,8,16, and $N_{r}=2$. In the figure, lines represent the analytical results obtained from Equations (8) and (22), while symbols represent link-level simulation results. The mathematical analysis derived in this paper matches well with the computer simulations over all SNR values. As *N* increases, the BER performance of the MUST-enhanced UE becomes improved due to the frequency diversity. Note that the lower bound of the average BER of the MUST-enhanced UE in Equation (22) is almost the same as the computer simulation especially when the SNR is high. The BER performance of the MUST-based UE is better than that of the MUST-enhanced UE in low SNR values due to the larger power allocation to the MST-based UE. On the other hand, it becomes worse than the BER of the MUST-enhanced UE for high SNR values due to the diversity gain of the MUST-enhanced UE.

**Figure 6:**
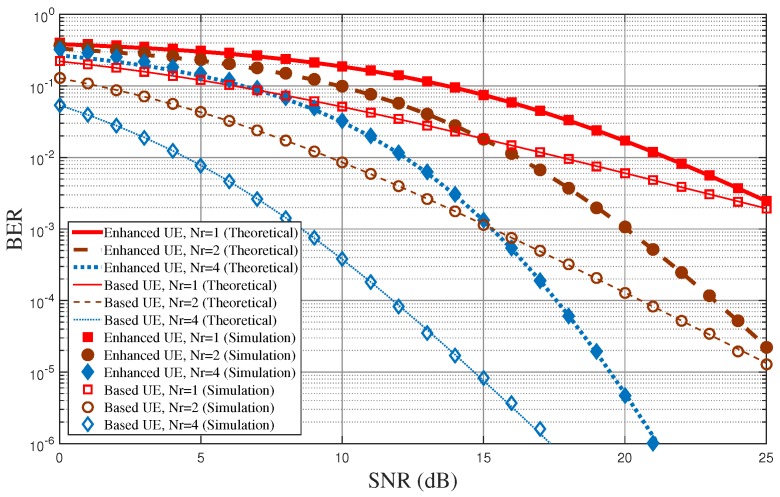
BER performance of the proposed MUST technique for varying SNR values when α=0.05, N=2, and $N_{r}=1,2,4$.

Figure 6 shows the BER performance of the proposed diversity-controlled MUST technique for varying SNR values when α=0.05, N=2, and $N_{r}=1,2,4$. Figure 5 shows the BER performance according to the number of frequencies used for the MUST-enhanced UE for a given number of receive antennas at UEs, but Figure 6 shows BER performance according to the number of receive antennas at UEs for a given number of frequencies used for the MUST-enhanced UE. The performance tendencies in both Figure 5 and Figure 6 are quite similar. The diversity order of the MUST-based UE is approximately equal to $N_{r}$, while that of the MUST-enhanced UE is approximately equal to $N\cdot N_{r}$.

**Figure 7:**
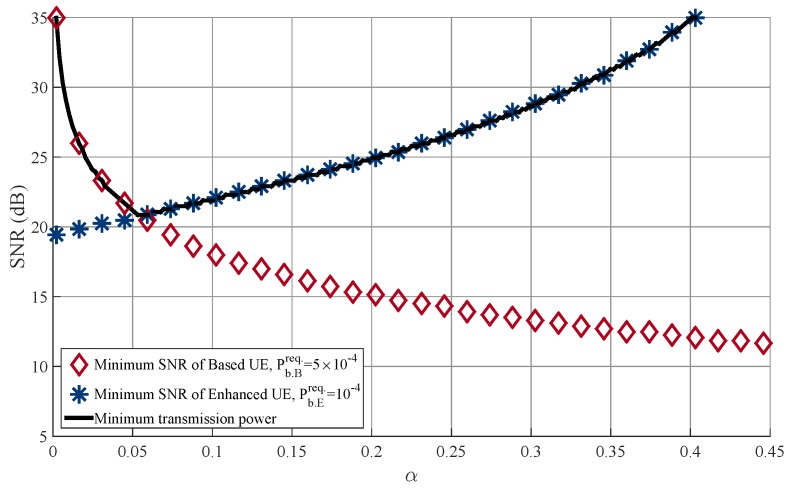
Required transmission power at BS according to α when N=2 and $N_{r}=2$.

Figure 7 shows the transmit SNR at the BS over varying α where the transmit power of the BS is adapted to satisfy the BER requirements when N=2 and $N_{r}=2$. We assume that $P_{b.B}^{req}=5\times 10^{-4}$ and $P_{b.E}^{req.}=10^{-4}$. As expected, the required SNR for the MUST-based UE decreases as α increases, while that for the MUST-enhanced UE increases as α increases. In this figure, the optimal α is equal to 0.06 and the minimum required SNR is equal to 21 dB.

## 6. Conclusions

In this paper, we proposed a generalized diversity-controlled MUST technique for 5G downlink cellular networks, which can be easily implemented with low complexity. We mathematically analyzed the BER performance of the proposed diversity-controlled MUST technique in Rayleigh fading environments where the BS is equipped with a single antenna but each user is equipped with multiple antennas. To the best of our knowledge, this is the first time that the closed-form solution on the BER of the diversity-controlled MUST technique has been obtained even though there are several link-level simulation results in the literature. The mathematical analysis was validated via extensive link-level simulations with various system parameters. Furthermore, based on the mathematical analysis, the optimal power allocation framework was considered to minimize the power consumption at the BS for given BER requirements. We leave the case of multiple antennas at the BS for further study.

## Figures and Tables

**Table 1:** Characteristics of MUST Techniques in 3GPP LTE Systems.

	Power Ratio of Signal	Bit-Labeling
MUST Category 1	adaptive	non-Gray mapping
MUST Category 2	adaptive	Gray mapping
MUST Category 3	N/A	Gray mapping

**Table 2:** Computation complexity of the receiver at the MUST-based UE.

	Number of Multipliers	Number of Adders	Number of Comparators
MUST-based UE	$N_{r}$	$N_{r}-1$	1

**Table 3:** Computation complexity of the receiver at the MUST-enhanced UE.

	Number of Multipliers	Number of Adders	Number of Comparators
MUST-enhanced UE	$2NN_{r}$	$2N(N_{r}-1)$	N+1
